# Supplementary material for: Multidisciplinary Consulting Team for Complicated Cases of Neurodevelopmental and Neurobehavioral Disorders: Assessing the Opportunities and Challenges of Integrating Pharmacogenomics into a Team Setting
Source: J Pers Med. 2022 Apr 8;12(4):599. doi: 10.3390/jpm12040599 (PMC9024886; doi:10.3390/jpm12040599)
Supplement: Supplementary file 1 [file jpm-12-00599-s001.zip › jpm-1604537-supplementary.pdf]

Journal of *Personalized Medicine*

MDPI

Article

Multidisciplinary Consulting Team for Complicated Cases of Neurodevelopmental and Neurobehavioral Disorders: Assessing the Opportunities and Challenges of Integrating Pharmacogenomics into a Team Setting

Pritmohinder S. Gill [1,2,*], Amanda L. Elchynski [3], Patricia A. Porter-Gill [2], Bradley G. Goodson [4], Mary Ann Scott [4], Damon Lipinski [4], Amy Seay [4,5], Christina Kehn [4], Tonya Balmakund [1,5] and G. Bradley Schaefer [1,2,4,6]

1 Department of Pediatrics, University of Arkansas for Medical Sciences, Little Rock, AR 72202, USA; balmakundtonyam@uams.edu (T.B.); schaefergb@uams.edu (G.B.S.)
2 Arkansas Children's Research Institute, Little Rock, AR 72202, USA; portergillpa@archildrens.org
3 Arkansas Children's Hospital, 1 Children's Way, Little Rock, AR 72202, USA; elchynskia@archildrens.org
4 Schmieding Developmental Center, Springdale, AR 72762, USA; bggoodson@uams.edu (B.G.G.); scottmar-yann@uams.edu (M.A.S.); dlipinski@uams.edu (D.L.); adseay@uams.edu (A.S.); kehnchristina@uams.edu (C.K.)
5 Arkansas Children's Hospital Northwest, Springdale, AR 72762, USA
6 University of Arkansas for Medical Sciences Northwest, Fayetteville, AR 72701, USA
* Correspondence: psgill@uams.edu; Tel.: +1-(501)-364-1418; Fax: +1-(501)-364-3654

Citation: Gill, P.S.; Elchynski, A.L.; Porter-Gill, P.A.; Goodson, B.G.G.; Scott, M.A.; Lipinski, D.; Seay, A.; Kehn, C.; Balmakund, T.; Schaefer, G.B. Multidisciplinary Consulting Team for Complicated Cases of Neurodevelopmental and Neurobehavioral Disorders: Assessing the Opportunities and Challenges of Integrating Pharmacogenomics into a Team Setting. *J. Pers. Med.* **2022**, *12*, 599. https://doi.org/10.3390/jpm12040599

Academic Editors: Angelos Halaris and José A. G. Agúndez

Received: 3 February 2022
Accepted: 6 April 2022
Published: 8 April 2022

Publisher's Note: MDPI stays neutral with regard to jurisdictional claims in published maps and institutional affiliations.

Copyright: © 2022 by the authors. Submitted for possible open access publication under the terms and conditions of the Creative Commons Attribution (CC BY) license (https://creativecommons.org/licenses/by/4.0/).

Abstract: Neurodevelopmental disorders have steadily increased in incidence in the United States. Over the past decade, there have been significant changes in clinical diagnoses and treatments some of which are due to the increasing adoption of pharmacogenomics (PGx) by clinicians. In this pilot study, a multidisciplinary team at the Arkansas Children's Hospital North West consulted on 27 patients referred for difficult-to-manage neurodevelopmental and/or neurobehavioral disorders. The 27 patients were evaluated by the team using records review, team discussion, and pharmacogenetic testing. OneOme RightMed® (Minneapolis, MN, USA) and the Arkansas Children's Hospital comprehensive PGx test were used for drug prescribing guidance. Of the 27 patients' predicted phenotypes, the normal metabolizer was 11 (40.8%) for CYP2C19 and 16 (59.3%) for CYP2D6. For the neurodevelopmental disorders, the most common comorbid conditions included attention-deficit hyperactivity disorder (66.7%), anxiety disorder (59.3%), and autism (40.7%). Following the team assessment and PGx testing, 66.7% of the patients had actionable medication recommendations. This included continuing current therapy, suggesting an appropriate alternative medication, starting a new therapy, or adding adjunct therapy (based on their current medication use). Moreover, 25.9% of patients phenoconverted to a CYP2D6 poor metabolizer. This retrospective chart review pilot study highlights the value of a multidisciplinary treatment approach to deliver precision healthcare by improving physician clinical decisions and potentially impacting patient outcomes. It also shows the feasibility to implement PGx testing in neurodevelopmental/neurobehavioral disorders.

Keywords: neurodevelopmental disorders; autism spectrum disorder; ADHD; pharmacogenomics; CYP2D6; CYP2C19; phenoconversion

Supplementary Material:

Table S1. List of commonly prescribed psychotropic medications and Cytochrome P450 (CYP) genes.

**Drug Category**	**Drug**	**Gene**
**SSRIs (Selective Serotonin Reuptake Inhibitors)**	Citalopram<br>Escitalopram<br>Fluvoxamine<br>Paroxetine<br>Sertraline	*CYP2C19*<br>*CYP2C19*<br>*CYP2D6*<br>*CYP2D6*<br>*CYP2C19*
**Tricyclic Antidepressants**	Amitriptyline<br>Clomipramine<br>Desipramine<br>Doxepin<br>Imipramine<br>Nortriptyline<br>Trimipramine	*CYP2D6, CYP2C19*<br>*CYP2D6, CYP2C19*<br>*CYP2D6*<br>*CYP2D6, CYP2C19*<br>*CYP2D6, CYP2C19*<br>*CYP2D6*<br>*CYP2D6, CYP2C19*
**SNRIs (Serotonin and Norepinephrine Reuptake Inhibitors)**	Venlafaxine	*CYP2D6*
**Antipsychotics**	Aripiprazole<br>Haloperidol<br>Risperidone	*CYP2D6*<br>*CYP2D6*<br>*CYP2D6*
**Anti-ADHD**	Atomoxetine	*CYP2D6*

Table S2 (A). *CYP2D6* and *CYP2C19* Star alleles (*) and rsID on the ACH-PGx Panel.

Current ACH PGx Panel-CYP2D6 & CYP2C19		
**Gene**	*** Allele or Common Variant Name**	**dbSNP or rs ID**
CYP2D6	multiple	rs1080985
CYP2D6	multiple	rs1135840
CYP2D6	*9	rs5030656
CYP2D6	*84	rs148769737
CYP2D6	*7	rs5030867
CYP2D6	*62	rs730882251
CYP2D6	*6	rs5030655
CYP2D6	*56A/B	rs72549347
CYP2D6	*54	rs267608297
CYP2D6	*53	rs1135823
CYP2D6	*51	rs72549348
CYP2D6	*49, *53	rs1135822
CYP2D6	*47	rs267608313
CYP2D6	*45, *46	rs28371710
CYP2D6	*44	rs72549349
CYP2D6	*43, *46	rs28371696
CYP2D6	*42	rs72549346
CYP2D6	*41, *69	rs28371725
CYP2D6	*4	rs3892097
CYP2D6	*38	rs72549351
CYP2D6	*35	rs769258
CYP2D6	*33	rs28371717
CYP2D6	*31	rs267608319
CYP2D6	*30, *40, *58	rs72549356
CYP2D6	*3	rs35742686
CYP2D6	*29,*70	rs61736512
CYP2D6	*29,*70	rs61736512adjC
CYP2D6	*29, *70	rs59421388
CYP2D6	*21	rs72549352
CYP2D6	*2, others	rs16947
CYP2D6	*19	rs72549353
CYP2D6	*18	hCV32407220
CYP2D6	*17, *40, *58	rs28371706
CYP2D6	*15	rs774671100
CYP2D6	*12	rs5030862
CYP2D6	*11	rs201377835
CYP2D6	*101	rs730882170
CYP2D6	*100	rs267608279
CYP2D6	*10, *36, others	rs1065852
CYP2D6	*14A,*14B,*8	rs5030865
CYP2C19	*17	rs12248560
CYP2C19	*35	rs12769205
CYP2C19	*9	rs17884712
CYP2C19	*4A;*4B	rs28399504

CYP2C19	*8	rs41291556
CYP2C19	*2	rs4244285
CYP2C19	*3	rs4986893
CYP2C19	*5	rs56337013
CYP2C19	*11	rs58973490
CYP2C19	*10	rs6413438
CYP2C19	*6	rs72552267
CYP2C19	*7	rs72558186

Table S2 (B). *CYP2D6* Copy Number Variation assay on ACH-PGx panel: (ThermoFisher Scientific, Carlsbad, CA, USA).

CYP2D6<br>Hs00010001_cn	Exon 9	*CYP2D6* Deletion (*5); Duplications (*1xN, *2xN, *4x2, *9x2,*10x2, *17xN, *35x2)
CYP2D6<br>Hs04502391_cn	Intron 6	*CYP2D6* Deletion (*5); Duplications (*1xN, *2xN, *4x2, *9x2, *10x2, *17xN, *35x2); 2D6/2D7 hybrid alleles with 2D7 exon 9 sequences (*36, *57, *83, *4N)
